# Influence of growth hormone receptor exon 3 polymorphism on growth response in children with growth hormone deficiency

**DOI:** 10.1186/1687-9856-2015-S1-P34

**Published:** 2015-04-28

**Authors:** Seung Yang, Il Tae Hwang

**Affiliations:** 1 Hallym University College of Medicine, Seoul, Republic of Korea

## Aims

Pharmacogenetic effects of recombinant human growth hormone according to growth hormone receptor (GHR) exon 3 polymorphism (fl vs. d3) were controversial. We investigated growth hormone response in children with growth hormone deficiency (GHD).

## Methods

Total 58 prepubertal children (31 boys and 27 girls) with GHD were enrolled in this study. Subjects were divided to 2 groups according polymorphism (fl/fl, n=48; fl/d3 and d3/d3, n=10), and compared baseline phenotypes and the first year growth response to growth hormone treatment.

## Results

The distribution of GHR exon 3 isoforms in children with GHD demonstrated that the frequency of fl/fl (82.8%) is higher than that in most of European studies. There was no significant difference in baseline height SDS between 2 groups. Height velocity during the first year of growth hormone replacement therapy tended to be higher in subjects who have d3 allele (fl/d3 and d3/d3), but there was no statistical difference according to genotype.

## Conclusion

It seemed that d3 allele of GHR exon 3 had no impact on the baseline phenotype and growth hormone response in patients with GHD. Relationship between GH dose and IGF-1% to help fully elucidate the value of IGF-1 testing in GH treatment.

